# Supplementary material for: Brain-controlled modulation of spinal circuits improves recovery from spinal cord injury
Source: Nat Commun. 2018 Aug 1;9:3015. doi: 10.1038/s41467-018-05282-6 (PMC6070513; doi:10.1038/s41467-018-05282-6)
Supplement: Supplementary file 1 — Supplementary Information [file 41467_2018_5282_MOESM1_ESM.pdf]

Supplementary Information

BRAIN–CONTROLLED MODULATION OF SPINAL CIRCUITS IMPROVES RECOVERY FROM SPINAL CORD INJURY

Bonizzato et al.

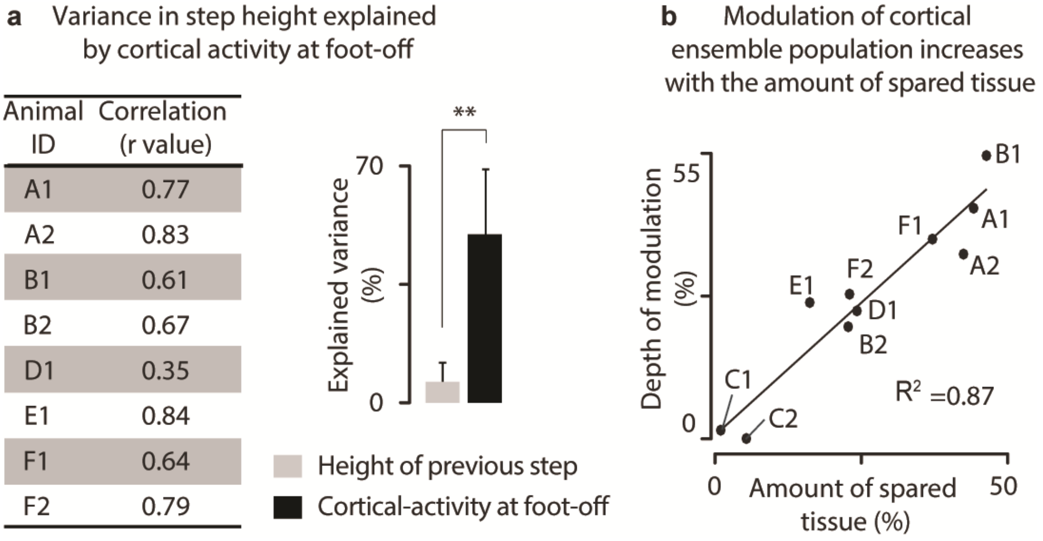

Supplementary Fig. 1. Modulation of cortical ensemble population correlates with step height, but depends on the amount of spared tissue. (a) For each rat, the correlation between the cortical activity at foot–off and the step height was calculated. The value of correlation coefficients is reported for each rat. The bar plot reports the mean variance of step height explained by the cortical activity at foot–off measured during the preceding step and for the ongoing step (n=8, mean ± SD). **, $P < 0.01$. **(b)** Correlation between the amount of spared tissue (%) and the extent of cortical population ensemble modulation during locomotion for all the experimental rats involved in the design of the brain–spine interface. The modulation is expressed in percent of increase of firing rate during locomotion compared to rest. The labels identifying each rat refer to **Supplementary Table 1**. Note that rats C1 and C2 were excluded from the study due to the absence of modulation in cortical ensemble population due to pronounced tissue damage (outside the targeted range of SCI severity).

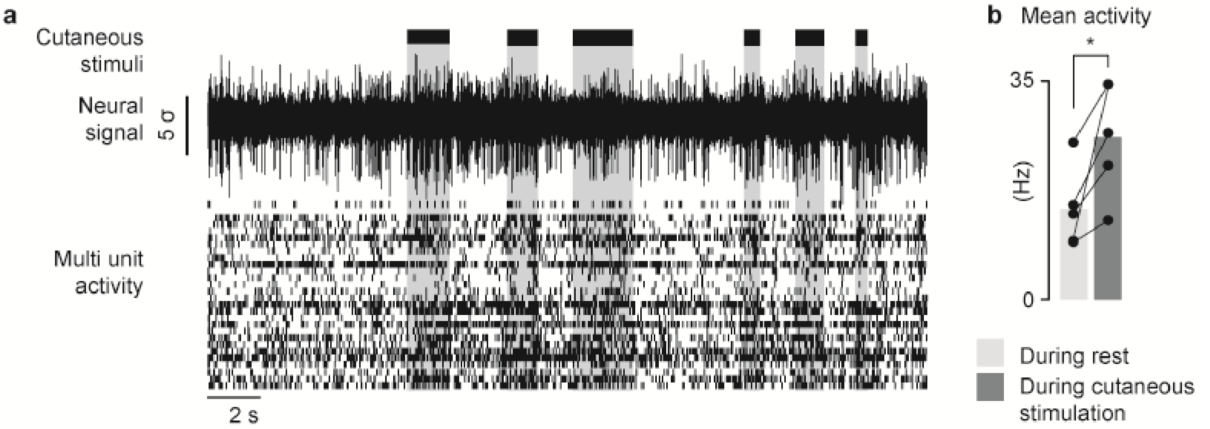

Supplementary Fig. 2. Cortical activity evoked by sensory stimulation of the paw. (a) Example of cortical activity (single channel and multi-unit activity) in response to successive applications of a pressure on the paw contralateral to the recordings. The horizontal bars and shaded region highlight the time windows over which the stimulation was applied. Recordings were performed at 3 weeks post-injury. **(b)** Bar plot reporting the mean activity measured over all the recorded multi-units during rest and over the period of cutaneous stimulation (n=5). *, $P < 0.05$.

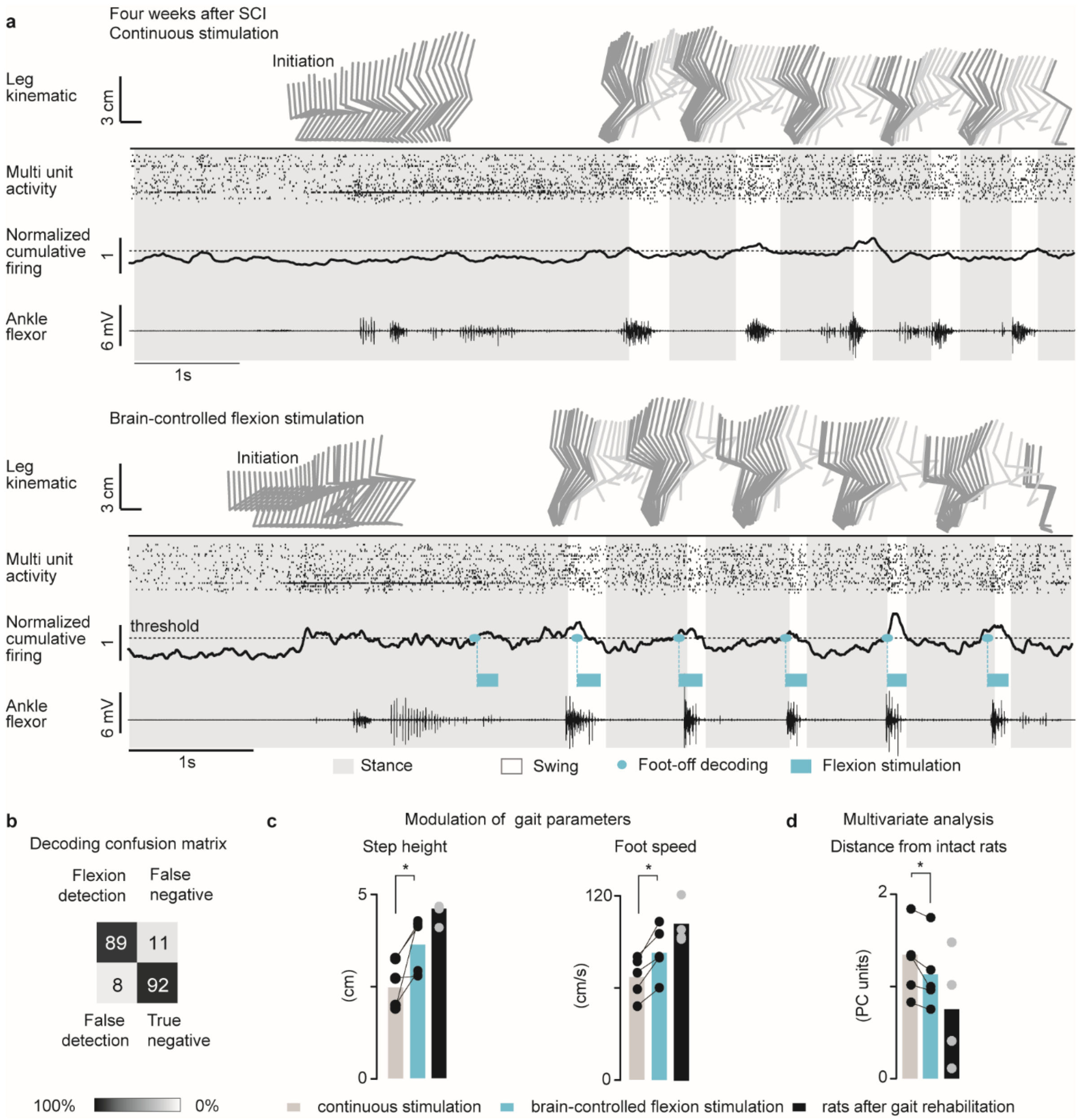

Supplementary Fig. 3. Binary brain–spine interface alleviates locomotor deficits during overground locomotion. (a) Recordings of bipedal locomotion along the runway at 3 weeks post–injury during continuous stimulation and with the binary brain–spine interface. Conventions are the same as in **Fig. 2**. **(b)** Confusion matrix of foot–off event decoding calculated across the 5 rats. **(c)** Bar plots reporting mean values and individual mean values of parameters modulated during continuous stimulation versus brain–controlled flexion stimulation. The values recorded in rats after gait rehabilitation are reported as a reference. **(d)** Bar plot reporting the distance from intact rats in the PC space calculated from 55 gait parameters, which thus quantifies locomotor performance. *, $P < 0.05$.

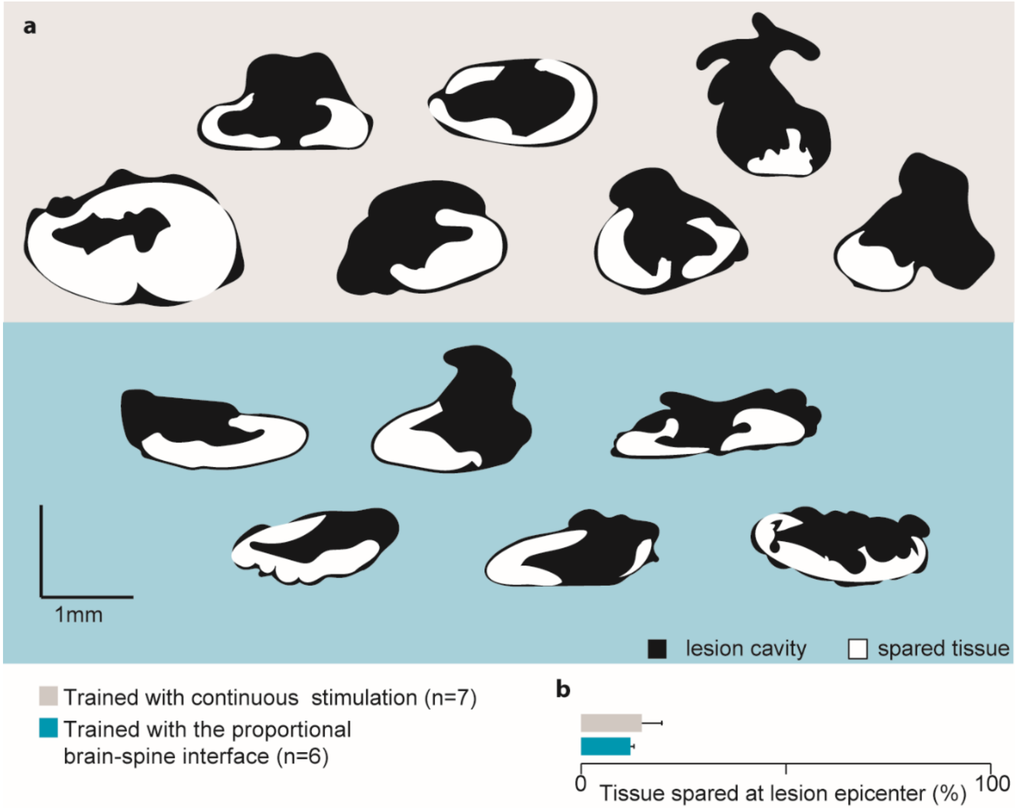

Supplementary Fig. 4. Quantification of the amount of spared spinal cord tissue. (a) Reconstruction of the lesion cavity (black) and spared tissue (white) at the epicentre of the contusion for both trained groups. **(b)** Bar plot reporting the amount of spared tissue for the two trained groups (mean ± SEM).

Animal ID	Binary BSI on treadmill Fig. 2	Binary BSI overground Supp. Fig. 3	Cortical modulation vs. spared tissue Supp. Fig. 1	Proportional BSI early after SCI Fig. 6	Rehabilitation enabled by proportional BSI Fig. 10	Proportional BSI staircase/overground Fig. 8	Cutaneous paw stimulation Supp. Fig. 2
A1	✓		✓				✓
A2	✓		✓				✓
B1		✓	✓				
B2		✓	✓				
C1			✓				
C2			✓				
D1			✓				
E1	✓	✓	✓				
F1	✓	✓	✓	✓			
F2	✓	✓	✓	✓			
G1				✓			
G2				✓			
G3				✓			
H1					✓		
H2					✓		
H3					✓		
H4					✓		
H5					✓		
H6					✓		
H7					✓		
I1					✓		
I2					✓	✓	
I3					✓	✓	
I4					✓	✓	
I5					✓	✓	
I6					✓	✓	
J1							✓
J2							✓
J3							✓

Supplementary Table 1. List of experimental procedures conducted per group of rats that participated to the experiments.

Temporal features of gait

1 Duration of gait cycle
2 Speed of animal during stride
3 Stance duration
4 Swing duration
5 Drag duration

Limb endpoint trajectory

6 Step height
7 Ankle clearance
8 Maximal foot speed during swing
9 Foot acceleration at swing onset
10 Foot speed at swing onset
11 Foot lateral displacement during swing

Stability

12 Stance width
13 Maximum hip vertical position
14 Minimum hip vertical position
15 Hip oscillation amplitude
16 Pelvic center of mass vertical motion

Joint angles

17 Hip joint excursion
18 Knee joint excursion
19 Ankle joint excursion
20 Hip joint speed depth
21 Knee joint speed depth
22 Ankle joint speed depth
23 Foot lateral oscillation

Segmental oscillations

24 Whole-limb excursion amplitude
25 Whole-limb lateral excursion amplitude
26 Whole limb speed depth
27 Crest elevation depth
28 Thigh elevation depth
29 Leg elevation depth
30 Foot elevation depth

Limb coordination

31 Temporal coupling between crest and thigh
32 Temporal coupling between thigh and leg
33 Temporal coupling between leg and foot
34 Correlation between crest and thigh oscillations
35 Correlation between crest and leg oscillations
36 Correlation between crest and foot oscillations
37 Correlation between thigh and leg oscillations
38 Correlation between thigh and foot oscillations
39 Correlation between leg and foot oscillations
40 Relative duration between crest and thigh angle minima
41 Relative duration between crest and leg angle minima
42 Relative duration between crest and foot angle minima
43 Relative duration between thigh and leg angle minima
44 Relative duration between thigh and foot angle minima
45 Relative duration between leg and foot angle minima
46 Phase of Crest maximal contraction
47 Phase of Hip maximal contraction
48 Phase of Knee maximal contraction
49 Phase of Ankle maximal contraction
50 Phase of Foot maximal contraction
51 Lag between crest and thigh maxima
52 Lag between thigh and leg maxima
53 Lag between leg and foot maxima

Robotic assistance required

54 Percentage of body weight supported
55 Robot vertical force

Supplementary Table 2. Analysis of kinematic activity: parameters computed for quantification.
