# Supplementary material for: Brain-controlled modulation of spinal circuits improves recovery from spinal cord injury
Source: Nat Commun. 2018 Aug 1;9:3015. doi: 10.1038/s41467-018-05282-6 (PMC6070513; doi:10.1038/s41467-018-05282-6)
Supplement: Supplementary file 3 — Description of Additional Supplementary Files [file 41467_2018_5282_MOESM3_ESM.pdf]

Description of Additional Supplementary Files

File Name: Supplementary Movie 1
Description: The first sequence shows the online decoding of foot-off events of the left leg (blue light) from cortical population responses during locomotion on a treadmill. The second sequence shows the enhancement of leg flexion when the detection of foot-off events (top blue light) triggers a burst of electrical stimulation over the spinal cord region involved in leg flexion. Movies are also shown in slow motion (5x).

File Name: Supplementary Movie 2
Description: The first sequence shows a rat walking overground with the proportional BSI. The second sequence shows the same rat during stair climbing with continuous stimulation and with the proportional BSI. The third sequence showed two rats tested with continuous stimulation during walking overground, but trained with continuous stimulation versus with proportional BSI.
